# Analysis of Transformation Plasticity in Steel Using a Finite Element Method Coupled with a Phase Field Model

**DOI:** 10.1371/journal.pone.0035987

**Published:** 2012-04-25

**Authors:** Yi-Gil Cho, Jin-You Kim, Hoon-Hwe Cho, Pil-Ryung Cha, Dong-Woo Suh, Jae Kon Lee, Heung Nam Han

**Affiliations:** 1 Department of Materials Science and Engineering and Center for Iron & Steel Research, RIAM, Seoul National University, Seoul, Republic of Korea; 2 Sheet Products and Process Research Group, Technical Research Laboratories, POSCO, Pohang, Republic of Korea; 3 School of Advanced Materials Engineering, Kookmin University, Seoul, Republic of Korea; 4 Graduate Institute of Ferrous Technology, Pohang University of Science and Technology, Pohang, Republic of Korea; Massachusetts Institute of Technology, United States of America

## Abstract

An implicit finite element model was developed to analyze the deformation behavior of low carbon steel during phase transformation. The finite element model was coupled hierarchically with a phase field model that could simulate the kinetics and micro-structural evolution during the austenite-to-ferrite transformation of low carbon steel. Thermo-elastic-plastic constitutive equations for each phase were adopted to confirm the transformation plasticity due to the weaker phase yielding that was proposed by Greenwood and Johnson. From the simulations under various possible plastic properties of each phase, a more quantitative understanding of the origin of transformation plasticity was attempted by a comparison with the experimental observation.

## Introduction

The transformation plasticity is believed to be a deformation mechanism that causes permanent deformation during the phase transformation of allotropic polycrystalline materials, even under an extremely small applied stress. For ideally plastic materials, Greenwood and Johnson [1] derived an analytical solution for permanent strain due to the transformation plasticity assuming that plastic deformation occurs in a weaker phase to accommodate the external and internal stresses caused by volume mismatch between two allotropic phases. The transformation plastic strain increment Δ*ε^tp^* under an uniaxial stress state was derived as follows:
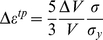
(1)where Δ*V*/*V* is the absolute value of the volume mismatch, and *σ* and *σ_y_* are the externally applied stress and uniaxial yield stress of the weaker phase, respectively.

Although their description is a widely accepted in the diffusional transformation plasticity, a later study by Zwigl and Dunand [2] showed that Greenwood and Johnson's derivation was valid only for small applied stresses compared to the yield stress. In their work [2], Greenwood and Johnson's theory was extended to relatively higher applied stress. However, the extended analytical solution could not provide any information on the internal or macroscopic strains that are dependent on time during a phase transformation. A couple of years later, they proposed a numerical model [3] that can generate time dependent information as well considering the temperature dependent properties of a material. The proposed model of transformation plasticity for an elastic, ideally plastic material was established through a two dimensional plane strain formulation considering both the temperature and displacement.

Recently, Greenwood and Johnson's model, so called the internal stress model, was elaborated theoretically as an explicit expression of the transformation plastic strain rate from the effort of Taleb and Sidoroff [4]. They improved the micro-mechanical model originally suggested by Leblond *et al*. [5] by removing some assumptions: elastic behavior of the product phase, and rigid plastic behavior of the parent phase. All these studies were based on conventional plasticity theory or the continuum mechanics.

Transformation plasticity is closely related to a phase transformation including interfacial movement, a morphologic construction, and other kinematical phenomena, of which combination eventually produces actual microstructure. However, the continuum-based theories have an obvious limitation in understanding the transformation plasticity because information on the microstructural evolution during phase transformation is absent. For this reason, in previous studies based on macroscopic conventional plasticity, the transformation plastic strain rate was adopted as an additional strain rate to reflect the additional plastic deformation caused by transformation plasticity.

Computer simulation methods have been effectively used to better understand phase transformations. The cellular automata (CA) method, for example, was used for the austenite-to-ferrite transformation in steels [6], [7]. The CA method simulates the impingement between newly formed grains well, but cannot take into account grain coarsening. A phase field model (PFM) has many advantages for the analysis of phase transformations comparing to the CA method. The PFM can handle grain coarsening and the impingement phenomenon, as well as consider diffusion, interface mobility, and the effect of interface energy [8]. Therefore, for the clearer understanding of transformation plasticity, it will be beneficial to incorporate microstructural information obtained from PFM into conventional continuum based theories, which have been suggested to interpret the transformation plasticity.

Many PFMs have been reported for the austenite-to-ferrite transformation. Yeon *et al*. [9] modified their phase field model for multicomponent alloy solidification [10] to describe the austenite-to-ferrite transformation of Fe-C-Mn ternary alloy under para-equilibrium. Mecozzi *et al*. [11] modified the model proposed by Steinbach *et al*. [12] and analyzed the microstructure evolution in the austenite-ferrite transformation of Fe-C-Mn alloy. Huang *et al*. [13] combined the solute diffusion model by Kim *et al*. [14] and the model proposed by Warren *et al*. [15] for multi-phase field and analyzed the microstructure evolution in the austenite-ferrite transformation of Fe-C binary alloy. Recently Cha *et al*. [16] proposed the PFM for the ferrite growth in the austenite poly-crystal including the effect of transformation stress.

In this study, we adopted the PFM proposed by Cha *et al*. [16] to describe the ferrite growth in the polycrystalline austenite microstructure. The effect of transformation stress, which requires expensive cost in the calculation, was not considered because our focus is not to develop rigorous PFM, rather than to investigate transformation plasticity associated with realistic evolution of microstructure. An implicit numerical solution procedure to calculate the deformation during the phase transformation of low carbon steel was implemented into the general purpose implicit finite element (FE) program. The procedure was coupled hierarchically with a PFM that could simulate the kinetics and microstructural evolution of the austenite-to-ferrite transformation, to evaluate the internal stress from volume mismatch between each phase involving the transformation. Therefore, any additional strain rate term for the transformation plastic deformation is not necessary to analyze the transformation plasticity. Only the thermo-elastic-conventional plastic constitutive equations for each phase were adopted to confirm the transformation plasticity due to the weaker phase yielding that was proposed by Greenwood and Johnson [1]. From the simulation, the origin of the transformation plasticity was discussed quantitatively in the context of Greenwood and Johnson's model [1] and compared with the other possible mechanisms [17]–[19].

## Methods

### Constitutive formulations

The Cauchy stress increment, *d* ***σ***, is
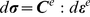
(2)where ***C*** *^e^* and *d* ***ε*** *^e^* are the elastic stiffness tensor and elastic strain increment, respectively. In the matter of elastic stiffness, isotropic elastic moduli [20] depending on the temperature were used, as listed in Table 1. Poisson's ratio of the steel was assumed to be constant, 0.3 [4]. The total strain increment, *d* ***ε*** *^T^* is
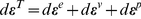
(3)where *d* ***ε*** *^v^* is the volumetric strain increment due to the phase transformation and temperature variation, and *d* ***ε*** *^p^* is the conventional plastic strain increment.

**Table 1:** Elastic modulus of steel at various temperatures [20].

Temperature, °C	500	700	800	900	1000	1100
Young's modulus, GPa	154	131	107	93	99	93

For the volumetric strain calculation, the linear mixture of strain increments of existing phases is assumed as follows:
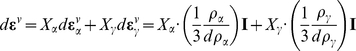
(4)where *X* and *ρ* are the phase fraction and density of each phase. The subscript *α* and *γ* mean the ferrite (*α*-iron) and austenite (*γ*-iron), respectively. ***I*** is identity tensor. The densities of austenite and ferrite were defined separately as a function of the temperature and chemical composition from Miettinen's data [21], as listed in Table 2.

**Table 2:** Densities of austenite and ferrite phase as a function of temperature and chemical composition [21].

Phase	Density, kg/m^3^
Austenite	8099.79-0.5060T+(−118.26+0.00739T)C_C_^γ^−68.24C_Si_^γ^−6.01C_Mn_^γ^
Ferrite	7875.96-0.2970T-5.62•10^−5^T^2^+(−206.35+0.00778T+1.472•10^−6^T^2^)C_C_^α^−36.86C_Si_^α^−7.24C_Mn_^α^
	T: temperature (in °C)C_M_^X^: content (in wt.%) of M in X phase

For the plastic strain increment, the flow rule can be written as follows under the von-Mises criterion.
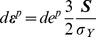
(5)where *de^p^* is the equivalent plastic strain increment, ***S*** is deviatoric stress, and *σ_Y_* is the yield stress. The yield condition is
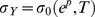
(6)where *σ_0_*(*e^p^*, *T*) is a function of equivalent plastic strain (*e^p^*) and temperature (*T*), which is defined for each phase. However, the reported data on the independent plastic behavior for each phase at a given temperature are found very rarely in literature, because the austenite and ferrite in low carbon steel generally coexist during the transformation. Here, based on the previous experimental data [22], it was assumed that the yield stresses of austenite and weaker ferrite phase are 100∼300 MPa and 80∼240 MPa with a linear work hardening rate of 8 MPa, respectively. The stress increment then becomes
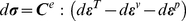
(7)The constitutive formulations were incorporated into the user material subroutine UMAT of ABAQUS/Standard [23], a commercial FE program.

### Phase field model

If we consider polycrystalline system where ferrite phase coexists with austenite phase, the governing equation of phase field model for austenite-to-ferrite transformation is [16]:
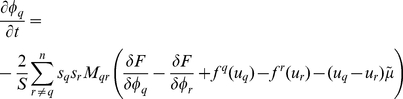
(8)where *α* and *γ* represent ferrite (*α*-iron) and austenite (*γ*-iron), respectively. The order parameter *φ_q_* (q = 1, 2, 3, …, n) gives the orientation state of a point in a polycrystalline system containing *n* grains, and the sum of all phase-field values in a point (i, j, k) is conserved as:
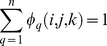
(9)If q>n/2, we define *φ_q_* as the orientation state of a point in the polycrystalline ferrite phase. Likewise, q<n/2 corresponds to austenite phase. A step function *s_q_* = 1, if *φ_q_*>0 and *s_q_* = 0 otherwise. The number of grains coexisting in a given point is 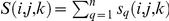. *M_qr_* is the phase field mobility. *u_q_* and *f^q^*(*u_q_*) are carbon concentration and the free energy density of *q* grain. 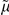 is the chemical potential of carbon, and we used the following constitutive equation [14], [16]:
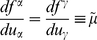
(10)The total free energy functional ***F*** includes the grain boundary (GB) or interphase boundary (IB) energy density *F^GB^* [16]:
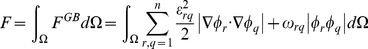
(11)The parameters *ω_rq_*, *ε_rq_*, and *M_qr_* in Eqs. (8) and (11) have the definite relationship with the GB or IB energy *σ_qr_* with its width 2*ξ_qr_* as follows:
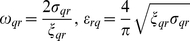
(12)and the mobility *M_qr_* is determined by the GB or IB mobility.

The governing equation of carbon diffusion is given as follows [16]:
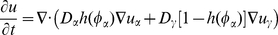
(13)where *D_α_* and *D_γ_* are the carbon diffusivities in the ferrite and austenite phases, respectively, and *φ_α_* is defined as the sum of the ferrite phase fields existing in a point, 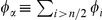. All points in the system are considered as the mixture of the ferrite and austenite phases and their fractions are given by *h*(*φ_α_*) and (1−*h*(*φ_α_*)), respectively. A function, *h*(*φ_α_*), is a monotonically increasing function for *φ_α_* between *h*(0) = 0 and *h*(1) = 1. The concentration, *u*, in the left-hand side of Eq. (13) is defined as
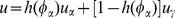
(14)Although various functions for *h*(*φ_α_*) can be used, this study employed the following function to minimize the solute trapping [24]

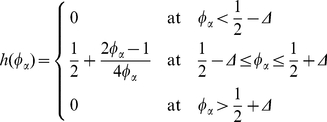
(15)The phase field mobility, *M_qr_*, is obtained from the interface mobility, *M_ij_* ^exp^, which is measured experimentally. The following relationship between *M_ij_* and *M_ij_* ^exp^ is obtained at a thin interface limit [25]:

for the ferrite/austenite interface:
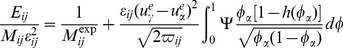
(16)where
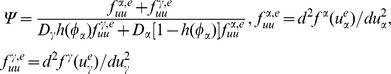
and for the ferrite and austenite grain boundaries
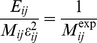
(17)


### Hierarchical multi-scale modeling: PFM into FEM

**Figure 1:**
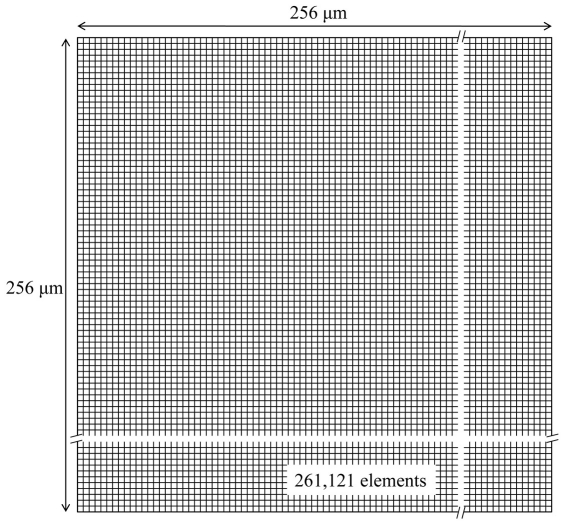
Two-dimensional square domain in PFM and FEM calculation.

**Figure 2:**
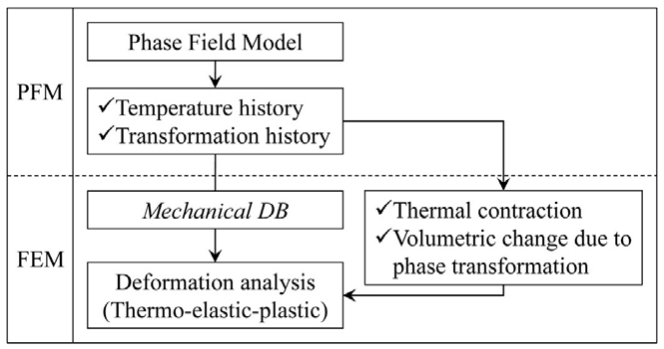
Flow of PFM-FEM hierarchical multi-scale simulation.

The PFM and finite element model (FEM) were established within a two-dimensional square shape domain, which has 256 µm on one side, as shown in Fig. 1. The domain is divided into 511×511 elements (or grids) in both calculations. This fine mesh system is needed because localized micro-stress field, which is caused by the volume change due to nucleation and growth of a newly formed phase, should be evaluated precisely to analyze the transformation plasticity. According to Greenwood and Johnson's model [1] and other previous studies [2]–[5], the localized micro-stress field can generate transformation plasticity, which originates from the micro-plasticity of the weaker phase. Fig. 2 shows the flow of a hierarchical multi-scale simulation that makes a connection between the PFM and FEM. Phase information for each element and each time step obtained from the PFM calculation is transferred into the FEM. However, for the temperature information, the temperature history in both cases is quite similar for the small calculation domain due to the relatively high conductivity of the steel. Therefore it is reasonable to assume a homogeneous temperature distribution within the entire domain.

**Figure 3:**
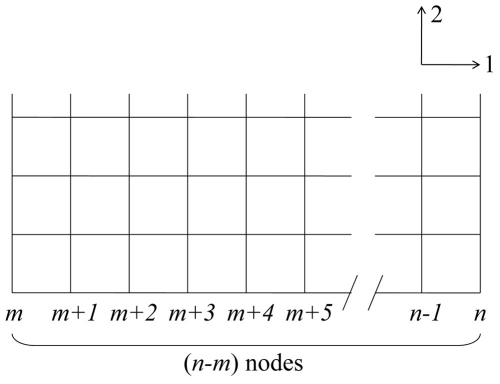
Schematic diagram of lower edge of FE calculation domain.

In the boundary conditions, the periodic boundary conditions (PBCs) should be adopted into the FE calculation to correspond to the PFM calculation. The unit cell size in the PFM is not changed during the calculation due to its finite difference scheme. However, the unit cell size in the FEM would be expanded or contracted according to the result of stress/displacement analysis. To be adequate requirements of PBCs in FEM, a linear multi-points constraint was applied to the boundaries of the FE domain. For example, let the lower edge of the domain in Fig. 1 be composed of (*n-m*) nodes (node number: *m*, *m+1*, …, *n-1*, *n*), as shown in Fig. 3. The linear multi-points constraint for the lower edge of the domain is as follows:
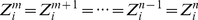
(20)where *Z* represents a nodal variable, the subscript means *i-*th degree of freedom, and the superscript means node number. In the above case, the *i*-th degree of freedom should be the displacement along the second axis.

The phase information for each position at each time step, which was calculated from PFM, was transferred to the corresponding element in the FEM that has exactly equivalent domain with PFM. In the PFM, the phase information is obtained as binary numbers, 0 or 1, for each node. Number 0 represents the austenite phase, and number 1 represents the transformed ferrite phase. Since the FE calculation of volumetric strain increment is performed on the Gaussian integration point of each element, not the nodal points, an interpolation technique for determining phase fraction was adopted in the FE calculation. The volumetric strain increment due to both phase transformation and temperature change was calculated in the FEM by using Eq. (4) and the densities as a function of temperature and chemical composition, which are listed in Table 2.

## Results and Discussion

### Hierarchical simulation for austenite-to-ferrite transformation

**Figure 4:**
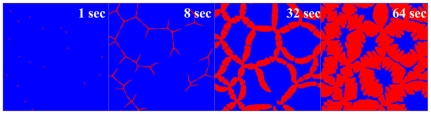
Calculated micro-structural evolution in PFM calculation. (Blue: Austenite, Red: Ferrite).

The evolution of austenite-to-ferrite transformation of low carbon steel was simulated using PFM assuming the chemical composition to be 0.003C-1.1 Mn (in wt.%). Steel with an initial temperature of 865°C was cooled continuously to 801°C with a cooling rate of 1°C/sec. The interface mobility between austenite and ferrite and the grain boundary mobilities of austenite and ferrite were assumed to be isotropic and to have the value obtained from the austenite/ferrite interface mobility proposed by Wits *et al.* [26]. The austenite/ferrite interface energy (γ_γα_) and grain boundary energies of austenite and ferrite are as follows: grain boundary energies of austenite and ferrite are 1.95 and 1 J/m^2^, respectively, and γ_γα_ = 1 J/m^2^. This combination of the interface and grain boundary energies produces ∼26 degrees of wetting angle. Due to the wetting angle, the ferrite phase nucleated on the grain boundary of austenite phase grows along austenite grain boundary like allotriomorph. It was assumed that the diffusion of Mn is negligible. Fig. 4 shows the calculated microstructures, which consist of initially full austenite and finally 80% ferrite with 20% austenite remaining. For validation of the calculated microstructures, the evolution of transformed microstructure was obtained experimentally. The quenching process, which means very rapid cooling, causes a diffusionless, martensitic transformation in the austenite remaining in the steel. This allows a measurement of the morphological features of ferrite and austenite during the transformation. The specimen for the experimental validation was 0.15C-1.4 Mn-0.25Si steels. The carbon content was designed to be slightly higher comparing to the PFM calculation to secure sufficient hardenability of the steel. Quenching after the small austenite-to-ferrite transformation was performed using a hot deformation simulator (THERMEC MASTER Z). The specimen was prepared as a cylindrical shape with 8 mm (Φ, diameter)×12 mm (length). The route of thermal processing is listed as follows:

- Heating to 1150°C at a heating rate of 5°C/sec
- Holding for 3 minutes
- Cooling to 700°C with a cooling rate of 2°C/sec
- Holding for 10, 20, 30 and 40 seconds
- Quenching with He gas

**Figure 5:**
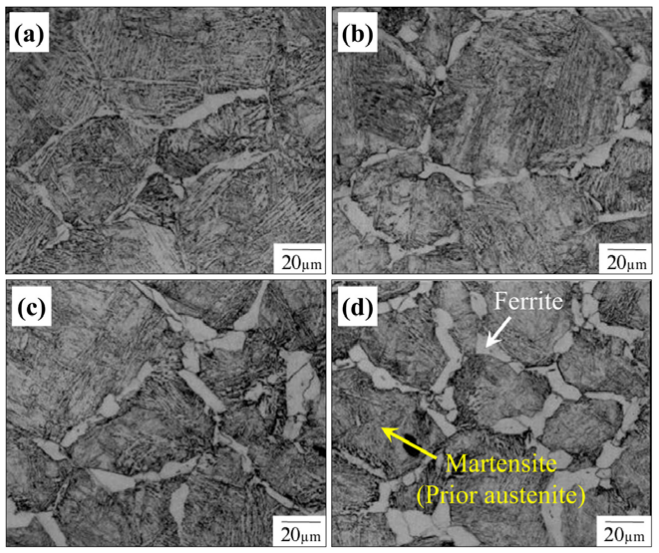
Optically observed microstructures (after quenching) during holding at 700°C. (a) 10 sec holding, (b) 20 sec holding, (c) 30 sec holding, and (d) 40 sec holding.

Fig. 5 shows the microstructures observed by optical microscopy. As shown in the figure, it could be considered that the microstructures calculated by PFM represent a comparable result to the experimentally observed one.

The calculated morphological evolution with PFM was transferred hierarchically into the FE calculation, which has an equivalent domain at the initial state. Thermo-elastic-plastic analyses were performed for the phase transformation of low carbon steel through the FE calculation. The following lists one of the calculation conditions:

- Yield stress of austenite: 200 MPa
- Slope of linear work hardening of austenite: 8 MPa
- Yield stress of ferrite: 160 MPa
- Slope of linear work hardening of ferrite: 8 MPa
- Externally uniaxial applied stress: 8 MPa along horizontal direction

**Figure 6:**
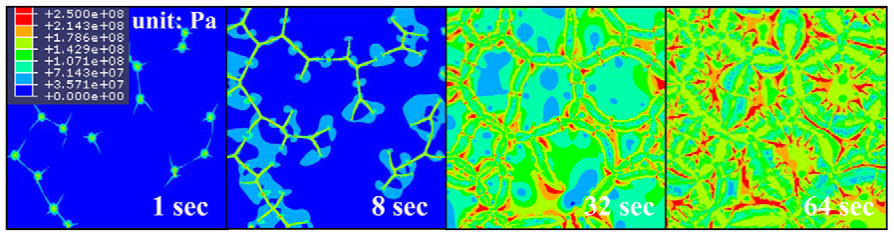
Calculated distribution of von-Mises stress during austenite-to-ferrite transformation.

**Figure 7:**
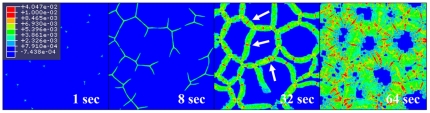
Calculated distribution of equivalent plastic strain during austenite-to-ferrite transformation. (arrow: front position of growing ferrite).

Fig. 6 shows the calculated distribution of von-Mises stress during the austenite-to-ferrite transformation of steel. The micro-stress field developed by the evolution of ferrite was calculated from FE analysis. Fig. 7 presents the distribution of equivalent plastic strain during the austenite-to-ferrite transformation. The evolution of micro-plasticity due to an externally and internally developed stress is concentrated mainly in the weaker ferrite phase. It is also found that the front position (see arrows in Fig. 7) of the growing ferrite grains receives relatively larger plastic deformation. This result appears to be in agreement with the internal stress model by Greenwood and Johnson [1], which suggests weaker phase yielding. However, micro-plastic deformation was also observed in the stronger austenite region, even though the amount of deformation is relatively small. It is possible that this result could be affected by the material properties used in the calculation, such as the hardening curves or yield stress ratio of ferrite to austenite. These effects will be discussed in the next section. From the above procedure, transformation plasticity, which might be caused by micro-plasticity in the weaker phase, can be evaluated without any additional terms for the transformation plastic strain with assistance of (a) accurate morphological data of the microstructure from the PFM calculation and (b) a very fine mesh system that is sufficient for application to the micro-scale phenomenon.

However, the quantitative amount of transformation plasticity could not be obtained directly from the calculated deformation, since the amounts of deformation along each side of the domain are not accurately equal to each other, even without external stress. In other words, the deformation of a domain could not be isotropic, even though there is no transformation plasticity. This means that the amount of non-isotropic deformation in this calculation does not match the amount of transformation plasticity directly. Moreover, the total amount of deformation contains not only the transformation plastic deformation but also the conventional plastic, thermal, and elastic deformation not relevant to the transformation plasticity. Here, the amount of transformation plasticity was evaluated as follows.

- Calculate the deformation in a reference state that represents the deformation without externally applied stress.
- Calculate the deformation under a non-zero externally applied stress with equivalent calculation conditions to the reference state.
- Subtract the reference deformation in (a) from the deformation in (b).

The elastic spring-back was also considered in the determination of transformation plasticity.

### Analysis of transformation plasticity

**Figure 8:**
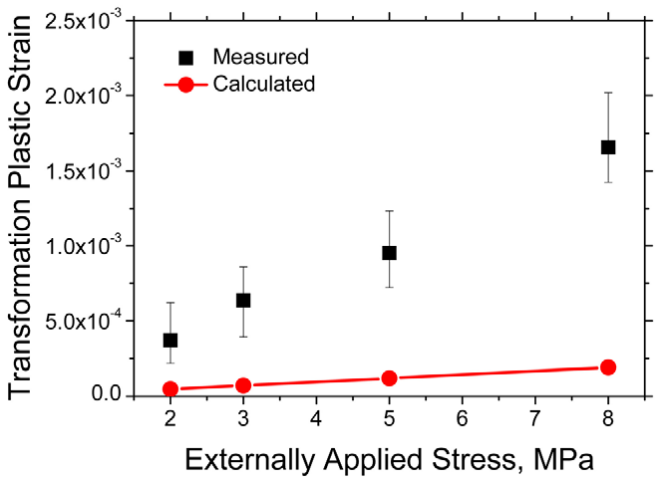
Comparison between measured [**18**] and calculated transformation plastic strains according to externally applied stress. (Yield stress of ferrite: 160 MPa, Yield stress of austenite: 200 MPa).

The principal factors that possibly affect the transformation plastic deformation in the present calculations can be classified into (a) an externally applied stress, (b) yield stress of each phase, and (c) yield stress ratio of ferrite to austenite. Initially, variations in the transformation plastic deformation with externally applied stresses along horizontal direction of the domain were evaluated. The applied stress was varied as 2, 3, 5 and 8 MPa, and the other calculation conditions, such as the yield stress of austenite and ferrite, and the cooling rate were the same as the calculation in the previous section. For a comparison, the experimental data in the previous study was adopted. The experimentally measured transformation plasticity has been reported in many studies [1], [19], [27]–[35]. A few years ago, some of the present authors [18] suggested a model for transformation plasticity in diffusional transformation with the experimental results during the austenite-to-ferrite transformation in plain low carbon steel. These experimental results are compared with the calculated results in the present study. Fig. 8 shows the measured [18] and calculated transformation plastic strains corresponding to externally applied stresses of 2, 3, 5 and 8 MPa. As expected from the internal stress model [1], the transformation plastic deformation was linearly proportional to the applied stress in both the measured and calculated cases. However, the measured amounts of transformation plastic deformation were significantly higher than the calculated ones. Since this disagreement might be caused by uncertainty in the mechanical properties of ferrite and austenite, several case studies were carried out under the conditions of the possible yield stress ranges of ferrite and austenite based on the previous experimental data [24].

**Figure 9:**
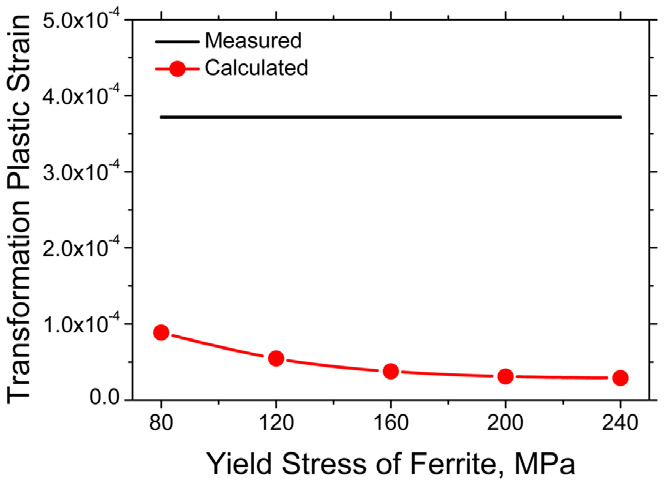
Measured [**18**] and calculated transformation plastic strain according to yield stress of ferrite. (Yield stress ratio of ferrite to austenite: 0.8, Externally applied stress: 2 MPa).

The amount of transformation plastic deformation varying with the yield stress of austenite and ferrite was evaluated in a consistent manner. The externally applied stress was fixed to 2 MPa. The yield stress of ferrite, which is assumed to be 80% of austenite strength, was varied as 80, 120, 160, 200 and 240 MPa. The calculated results are represented in Fig. 9. As shown in the figure, the amount of deformation due to transformation plasticity is in inverse proportion to the yield stress of ferrite. This tendency is also consistent with the internal stress model of Eq. (1), which suggests that the strain rate due to transformation plasticity is inversely proportional to the yield stress of the weaker phase. Another interesting point is that the transformation plasticity obtained under the condition of the extremely lowest yield stress of ferrite was still about over 4 times smaller than the measured one.

**Figure 10:**
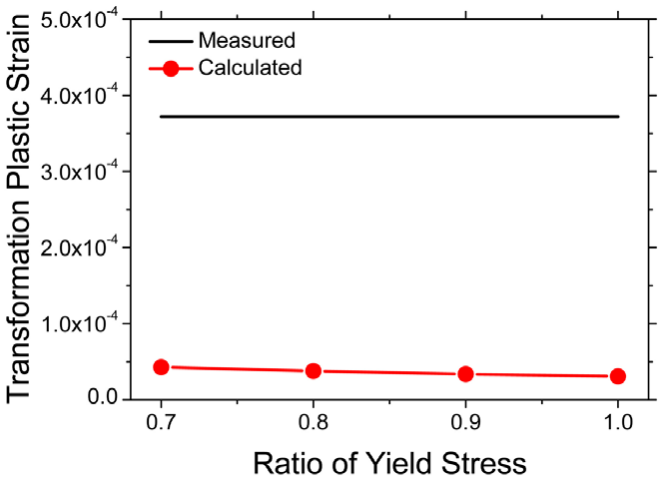
Measured [**18**] and calculated transformation plastic strain according to yield stress ratio of ferrite to austenite. (Yield stress of austenite: 200 MPa, Externally applied stress: 2 MPa).

**Figure 11:**
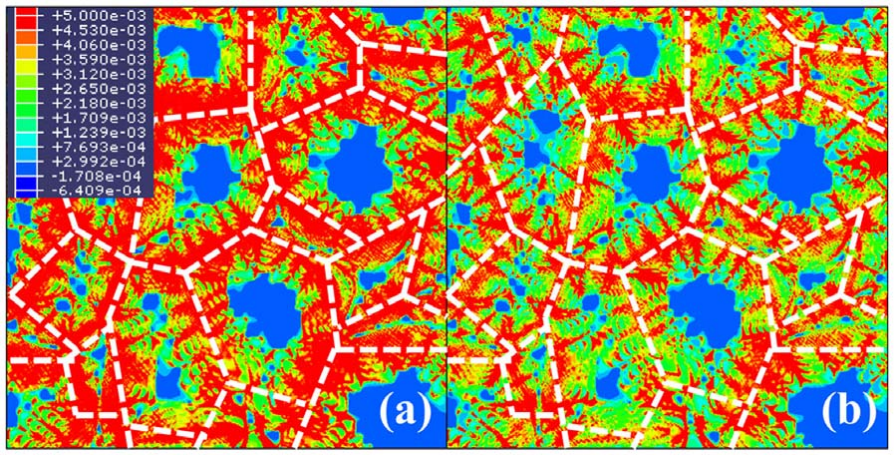
Calculated distribution of equivalent plastic strain for (a) 0.7 yield stress ratio of ferrite to austenite, and (b) 1.0 yield stress ratio. White dash line represents prior austenite grain boundary.

Lastly, the amount of transformation plastic deformation varying with the yield stress ratio of ferrite to austenite was obtained from FE calculations. In this case, the externally applied stress was fixed to 2 MPa, and the yield stress of austenite was fixed to 200 MPa as well. The yield stress of ferrite was changed as 140, 160, 180, and 200 MPa, which correspond to the yield stress ratio of 0.7, 0.8, 0.9, and 1.0, respectively. Fig. 10 shows the calculated transformation plasticity according to the yield stress ratio of ferrite to austenite. As the yield stress of ferrite approaches that of austenite, the transformation plastic deformation decreases because (a) the micro-plastic deformation by the evolution of phase transformation would be distributed more uniformly into both phases and (b) the yield stress of ferrite is increased. Fig. 11 shows the difference in the equivalent plastic strain distribution for the two extreme cases in these calculations, 0.7 and 1.0 yield stress ratio. The figure also shows that plastic deformation occurs in both stronger austenite phase and weaker ferrite phase. Indeed, the plastic deformation in the stronger austenite phase always occurs in all the calculations in this study. Therefore, the micro-plastic deformation during the phase transformation is not confined to the weaker phase, which is in contrast to that suggested by Greenwood and Johnson [1].

From Figs. 8, 9 and 10, it might be confirmed that only the conventional plasticity, which is caused by the volume mismatch between the prior and resultant phase, might not be sufficient to explain the transformation plastic deformation experimentally observed. In other words, the transformation plasticity might require the other mechanisms in addition to the Greenwood and Johnson's one. Indeed, Han *et al.* [36], [37] reported that considerable permanent strain was observed in extra low carbon steel during the recrystallization and growth under a small applied stress, even much lower than the yield stress. Since there is little volume mismatch in recrystallization and growth, the model based on the internal stress model by Greenwood and Johnson [1] is difficult to explain the above permanent deformation. Therefore, they suggested a model based on a migrating interface diffusion mechanism [18], [37], which was formulated as an accelerated Coble creep, to explain the permanent deformation due to both the phase transformation and recrystallization/growth. This model was confirmed recently by Cho *et al*. [38] for the case of various heat treatments on low and ultra low carbon steels. They incorporated this model into an implicit FEM, then showed the simulated results were in good agreement with the observed deformation behaviors.

As another possible mechanism for transformation plasticity, the selection of the specific variant during the phase transformation under external stress could be considered. In this mechanism, the preferentially selected variant with the specific orientation relationship between the variant and the applied stress field could lead to the occurrence of transformation plasticity [17], [19], [27].

### Conclusion

To calculate the deformation behavior during the phase transformation of low carbon steel, the numerical procedure was coupled hierarchically with PFM and FEM. PFM could simulate the kinetics and micro-structural evolution during the austenite-to-ferrite transformation. Phase information for each element and each time step obtained from the PFM calculation was transferred into the thermo-elastic-plastic FEM. From the developed method, it was confirmed that the transformation plasticity could be caused by a conventional plastic deformation of the weaker phase, which was suggested as the internal stress model by Greenwood and Johnson [1].

Through the case studies, the quantitative amounts of deformation due to the transformation plasticity versus (a) the externally applied stress, (b) the yield stress of weaker ferrite, (c) and the yield stress ratio of ferrite to austenite were presented, and the tendency of the transformation plastic deformation was confirmed with the one predicted by the internal stress model. However, the calculation results suggest that plastic flow is not confined to the weaker ferrite phase, which is unlike that suggested by the internal stress model. Rather, it occurs in both the weaker and stronger phases.

The important result is that the calculated amount of transformation plastic deformation was much less than the measured transformation plastic deformation. This difference suggests the possibility that the transformation plasticity cannot be fully understood only with the micro-plastic deformation of the weaker phase and the other mechanisms such as the accelerated Coble creep [18] or the specific variant selection [17], [19], [27] during the phase transformation may work simultaneously.

A limit of this study is related mainly with a fundamental restriction of continuum mechanics. Although we obtain several important results that overcome the conventional continuum approaches with an assistance of PFM, the atomistic phenomena such as the accelerated creep or the variant selection cannot be considered essentially in this FE based scheme. We believe that lower scale simulations such as molecular dynamics or ab-initio calculation, which describe the atomistic motion directly, would be helpful for more fundamental understanding to the transformation plasticity.
